# Successful Implementation of a Packed Red Blood Cell and Fresh Frozen Plasma Transfusion Protocol in the Surgical Intensive Care Unit

**DOI:** 10.1371/journal.pone.0126895

**Published:** 2015-05-26

**Authors:** Benjamin E. Szpila, Tezcan Ozrazgat-Baslanti, Jianyi Zhang, Jennifer Lanz, Ruth Davis, Annette Rebel, Erin Vanzant, Lori F. Gentile, Alex G. Cuenca, Darwin N. Ang, Huazhi Liu, Lawrence Lottenberg, Peggy Marker, Marc Zumberg, Azra Bihorac, Frederick A. Moore, Scott Brakenridge, Philip A. Efron

**Affiliations:** 1 Department of Surgery, University of Florida College of Medicine, Gainesville, FL, 32610, United States of America; 2 Department of Anesthesiology, University of Florida College of Medicine, Gainesville, FL, 32610, United States of America; 3 Department of Nursing, University of Florida College of Medicine, Gainesville, FL, 32610, United States of America; 4 Department of Medicine, University of Florida College of Medicine, Gainesville, FL, 32610, United States of America; 5 Department of Anesthesia, University of Kentucky College of Medicine, Lexington, KY, 40506, United States of America; 6 Department of Surgery, University of South Florida, Tampa, FL, 33612, United States of America; FDA, UNITED STATES

## Abstract

**Background:**

Blood product transfusions are associated with increased morbidity and mortality. The purpose of this study was to determine if implementation of a restrictive protocol for packed red blood cell (PRBC) and fresh frozen plasma (FFP) transfusion safely reduces blood product utilization and costs in a surgical intensive care unit (SICU).

**Study Design:**

We performed a retrospective, historical control analysis comparing before (PRE) and after (POST) implementation of a restrictive PRBC/FFP transfusion protocol for SICU patients. Univariate analysis was utilized to compare patient demographics and blood product transfusion totals between the PRE and POST cohorts. Multivariate logistic regression models were developed to determine if implementation of the restrictive transfusion protocol is an independent predictor of adverse outcomes after controlling for age, illness severity, and total blood products received.

**Results:**

829 total patients were included in the analysis (PRE, n=372; POST, n=457). Despite higher mean age (56 vs. 52 years, p=0.01) and APACHE II scores (12.5 vs. 11.2, p=0.006), mean units transfused per patient were lower for both packed red blood cells (0.7 vs. 1.2, p=0.03) and fresh frozen plasma (0.3 vs. 1.2, p=0.007) in the POST compared to the PRE cohort, respectively. There was no difference in inpatient mortality between the PRE and POST cohorts (7.5% vs. 9.2%, p=0.39). There was a decreased risk of urinary tract infections (OR 0.47, 95%CI 0.28-0.80) in the POST cohort after controlling for age, illness severity and amount of blood products transfused.

**Conclusions:**

Implementation of a restrictive transfusion protocol can effectively reduce blood product utilization in critically ill surgical patients with no increase in morbidity or mortality.

## Background

Evidence demonstrates blood product transfusions adversely affect patient outcomes. This is especially true in trauma and critically ill surgical patients, in whom it is associated with increased morbidity and mortality [1–3]. In fact, randomized controlled trials illustrate worsened outcomes with packed red blood cell (PRBC) transfusion in certain subsets of ICU populations [4–5]. These associations have led to the implementation of restrictive policies for transfusion in many hospitals in an attempt to improve outcomes in ICU patients [6].

Despite the known risks of blood product transfusion, 14 million units of PRBC are transfused annually in the United States [7]. Forty five percent of ICU patients receive blood product transfusions, which can increase to 85% depending on the patient’s length of stay [8–10]. In addition, the age of the stored product is associated with worsening outcomes; the average age of transfused PRBCs in the United States is 17 days old, and 20% of all transfused blood products are greater than 28 days old [8–10]. In previous studies, blood that was greater than or equal to 21 days, which is considered old blood, was shown to lead to decreased peripheral tissue oxygenation [11].

We hypothesized that a restrictive protocol for PRBC and fresh frozen plasma (FFP), when successfully instituted in a surgical intensive care unit (SICU), could significantly lower blood product utilization without an adverse effect on morbidity and mortality.

## Methods

Research was approved by the University of Florida IRB (IRB#6252011). Informed consent was not needed as all data was analyzed anonymously.

### Protocol implementation

A transfusion protocol with restrictive PRBC and FFP transfusion parameters was created and implemented in a surgical and trauma intensive care unit (SICU) at UF Health Shands Hospital at the University of Florida. This unit admits critically ill trauma, acute care general surgery, vascular, orthopedic and traumatic neurosurgery patients.

**Fig 1:**
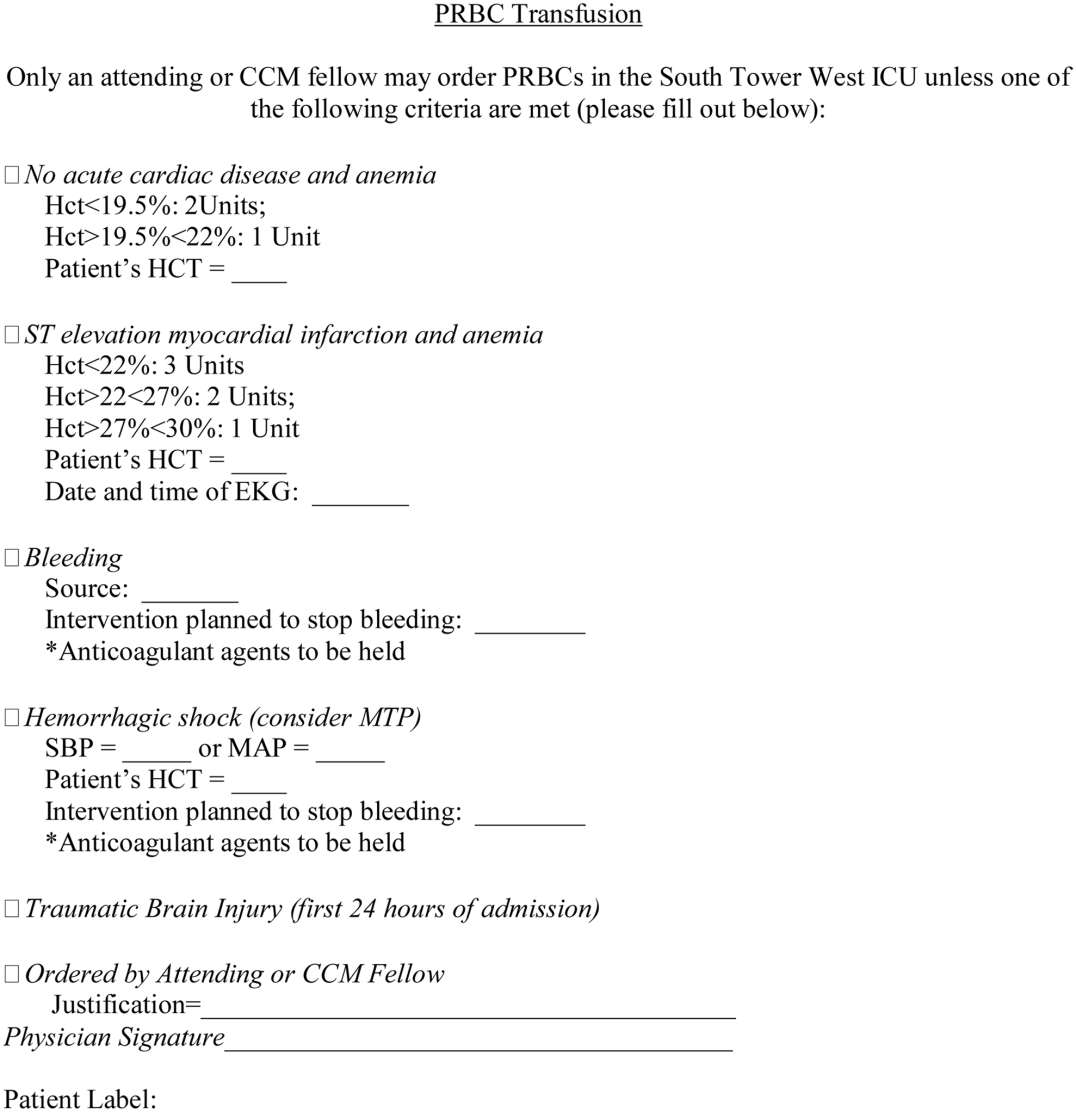
Restrictive transfusion protocol for PRBC.

**Fig 2:**
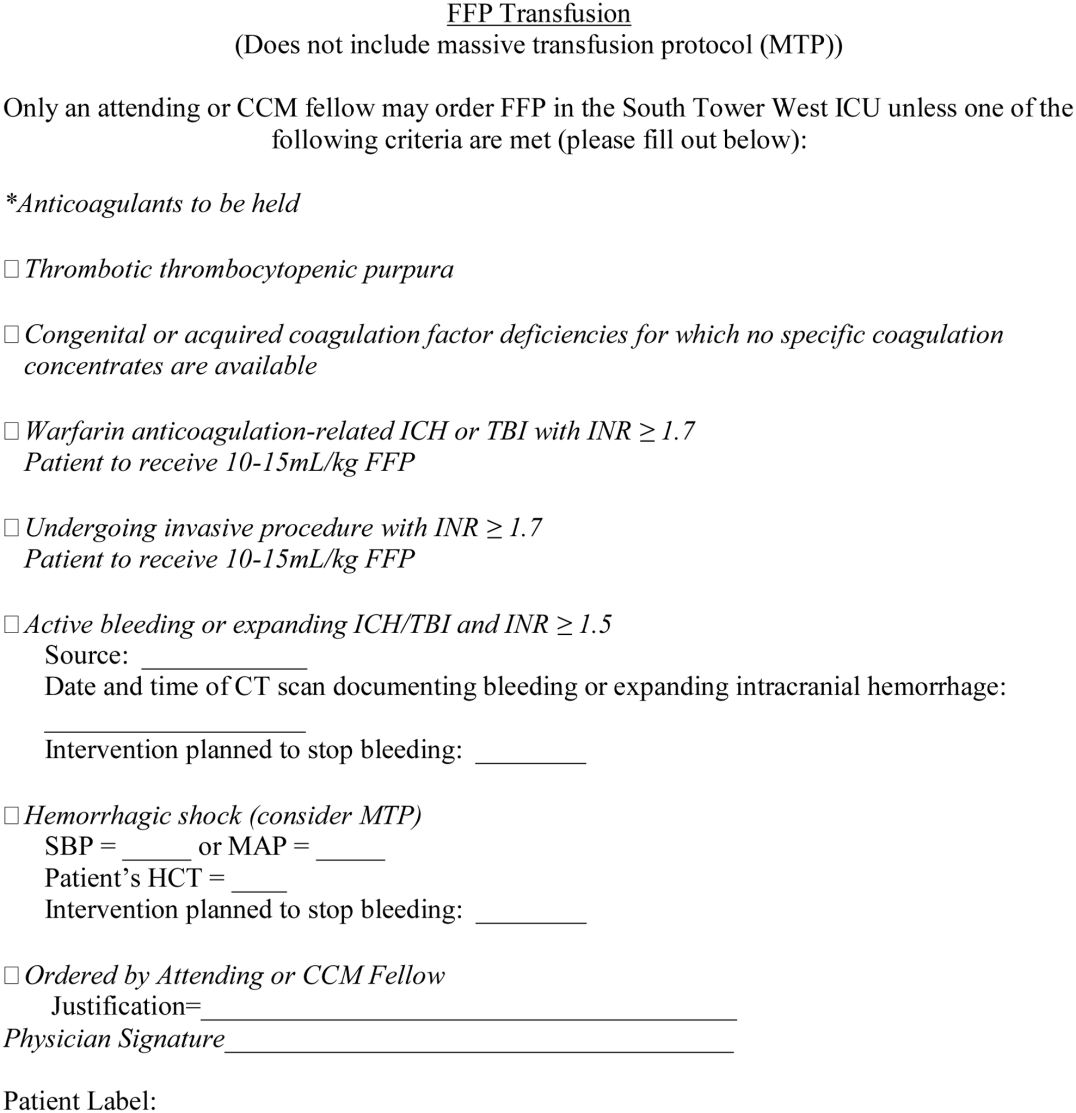
Restrictive transfusion protocol for FFP.

Resident physicians and advanced practitioners were allowed to transfuse PRBC and FFP only if patient parameters were consistent with the restrictive protocol (Figs 1 and 2). Surgical Critical Care (SCC) attending physicians and fellows (defined as critical care residents by the Accreditation Council for Graduate Medical Education) could order PRBC or FFP outside the listed criteria, but required justification and documentation of their reasoning. Nursing staff were trained to administer blood products only if consistent with the outlined protocol criteria as documented by a physician completed written form. Verbal orders for product transfusion were not allowed, except for emergent circumstances, as deemed by the attending surgeon, critical care fellow or SCC attending physician. Transfusion of blood products was recorded via the institution’s electronic medical record system (EPIC; Verona, WI.). Whether or not transfusion was under the auspices of the institutional Massive Transfusion Protocol (MTP) was also recorded. Our institutional MTP is enacted when there is a need for emergent transfusion in an adult patient, usually presumed to be 10 units of PRBCs or greater. The restrictive protocol was not applicable to transfusions performed in patients with confirmed active hemorrhage and/or during the MTP as these individuals are most often in hemorrhagic shock and therefore not appropriate for blood product restriction. The restrictive protocol was reinstituted in these patients once the MTP was no longer active. The units transfused during MTP were not counted towards the total units given; however, these patients were included once they were stabilized and could take part in the normal protocol.

Prior to implementation, the protocol was approved by the department and/or division chief of all relevant admitting surgical services. These criteria were then circulated to all admitting attending surgeons for review. This was followed by a concerted effort for ongoing education targeting surgeons, SICU physicians and advanced practitioners about best practices for blood product utilization in critically ill patients, including, but not limited to departmental “Grand Rounds” seminars and other didactic sessions. Additionally, nursing education was conducted, using a combination of presentations, email notifications and on-line didactic education.

### Data Source and Study Population

The two time periods that were analyzed include the four months following institution of the protocol (POST; March-June, 2011) as well as a historical control period (PRE; March-June, 2010). Data were obtained from the institutional electronic medical record, including patient demographics, severity of critical illness, blood product administration, complications and clinical outcomes. Number of operative procedures was determined based on total cases performed in a dedicated operative suite. For patients included in the study, the number of units transfused was only calculated if they were currently in the SICU.

### Statistical Analysis

Categorical variables were reported as frequencies and percentages and continuous variables were reported as means with standard deviations. Pearson χ^2^ test or Fisher’s exact test was used to test independence between categorical variables as appropriate. Sample size was large enough for valid application of the central limit theorem; therefore we utilized unpaired t-test for comparison of continuous variables. Outcomes were determined in a retrospective fashion using ICD-9 coding from clinical documentation. For variables achieving statistical significance with univariate testing, we constructed additional multivariable logistic regression models for each binary outcome using exposure group (POST vs. PRE) as the main predictor in order to adjust for age, gender, APACHE II score, ICU LOS, and total amount of blood products transfused. Adjusted odds ratios of having adverse outcome for POST compared to PRE cohorts were reported along with their 95% confidence intervals (CI). All significance tests were two-sided, with an α level of 0.05 to denote statistical significance. Statistical analyses were performed with SAS (v.9.3, Cary, N.C.).

## Results

A total of 839 patients were admitted to the SICU over the two time periods. 10 patients that did not have enough data in their chart to calculate their APACHE II score were excluded, leaving 829 patients for inclusion in the final analysis (n = 457 POST; n = 372 PRE). Univariate analysis of the PRE and POST cohorts revealed similar patient and clinical demographics, with the exception of the slightly increased age and APACHE II score in the POST group (both p<0.05) (Table 1). Admission INR levels, lowest hemoglobin in the first 24 hours of admission, total number of operative procedures, gender, and MTP activation were not significantly different between the two groups (Table 1). The total numbers of combined PRBC and FFP units transfused during the two time periods were 454 and 881 in the POST and PRE cohort, respectively. The mean per patient units transfused for PRBC (0.7 vs. 1.2, p = 0.03) and FFP (0.3 vs. 1.2, p = 0.007) units were lower in the POST compared to the PRE cohort (Table 2). This represents a 41% reduction of PRBC and 75% reduction of FFP units transfused per patient, adjusted for patient volume differences, between the PRE and POST periods. After excluding MTP patients, univariate analysis suggested a significantly higher rate of transfusion within the first 24 hours of admission to the SICU in the PRE cohort (Table 2). This association remained statistically significant after controlling for age and APACHE II scores, with a 47% decrease in odds of transfusion within 24 hours (Table 3). Preventable outcomes were defined as adverse events that were a direct result of failure to follow best practice measures or established guidelines.

**Table 1:** Demographics—Pre vs. Post Restrictive Transfusion Protocol.

	PRE		POST		
	(n = 372)		(n = 457)		
	Mean	SD	Mean	SD	(p)
Age (y)	52.5	(20.3)	56.0	(18.3)	0.01
APACHE II score	11.2	(7.1)	12.5	(7.0)	0.006
Admission PT^a^	16.1	(7.6)	16.5	(5.1)	0.39
Admission INR^b^	1.3	(0.6)	1.3	(0.6)	0.17
Lowest Hgb 1st 24 hrs^c^	10.8	(2.2)	10.5	(2.2)	0.054
Operative procedures^d^	1.1	(1.9)	1.3	(1.5)	0.10
	(n)	(%)	(n)	(%)	
Male gender	228	(61.3)	271	(59.3)	0.56
Massive Transfusion Protocol	15	(4.0)	22	(4.8)	0.59

Abbreviations: APACHE, Acute Physiology and Chronic Health Evaluation; PT, prothrombin time; INR, international normalized ratio; Hgb, hemoglobin; SD, standard deviation.

All percentages were calculated as column percentages.

^a^ Pre n = 327, Post n = 390;

^b^ Pre n = 326, Post n = 390;

^c^ Pre n = 370, Post n = 456;

^d^ Post n = 456;

**Table 2:** Patient Outcomes—Pre vs. Post Restrictive Transfusion Protocol.

	PRE		POST		
	(Mean)	(SD)	(Mean)	(SD)	(p)
Ventilator Days	3.9	10.7	2.9	6.5	0.11
ICU LOS	6.2	13.1	5.5	8.2	0.36
Hospital LOS	11.7	18.1	11.2	12.6	0.65
Units PRBC transfused^1^	1.2	(4.2)	0.7	(1.8)	0.034
Units FFP transfused^1^	1.2	(6.2)	0.3	(1.4)	0.007
	(n)	(%)	(n)	(%)	
Transfusion 1st 24 hrs (non-MTP)	44	11.8	35	7.7	0.04
Complications—Bleeding	12	3.2	21	4.6	0.32
Complications—Pneumonia	6	1.6	9	1.97	0.70
Complications—UTI	41	11.0	29	6.4	0.016
Complications—MI	9	2.4	11	2.4	0.99
Complications—Sepsis	35	9.4	52	11.4	0.36
Complications—PE	6	1.6	9	1.9	0.70
Complications—AKI	13	3.49	23	5.0	0.28
In-hospital mortality	28	7.5	42	9.2	0.39

Abbreviations. ICU, Intensive care unit; LOS, length of stay; PRBC, packed red blood cells; FFP, fresh frozen plasma; MTP, massive transfusion protocol; UTI, urinary tract infection; MI, myocardial infarction; PE, pulmonary embolus; AKI, acute kidney injury; SD, standard deviation.

^1^Mean units per patient.

**Table 3:** Multivariate Analysis of early transfusion and urinary tract infection rates.

Transfusion within 1st 24 hours (non-MTP)			
	Odds Ratio	95% CI	p
Restrictive Transfusion Protocol	0.53	0.33–0.86	0.011
APACHE II Score	1.10	0.07–1.14	<0.0001
**Urinary Tract Infection**			
	Odds Ratio	95% CI	p
Restrictive Transfusion Protocol	0.47	0.28–0.80	0.005
Age	1.028	1.01–1.04	0.0002
APACHE II Score	1.05	1.02–1.09	0.005
Units PRBC Transfused	1.15	1.05–1.25	0.003
Units FFP Transfused	0.90	0.79–1.02	0.09

Abbreviations. RR, relative risk; CI, confidence interval.

Abbreviations. APACHE, Acute Physiology and Chronic Health Evaluation; PRBC, packed red blood cells; FFP, fresh frozen plasma; CI, confidence interval.

There was no evidence of significant differences among inpatient outcomes between the PRE and POST cohorts, including preventable hospital mortality, bleeding, pneumonia, myocardial infarctions, pulmonary embolisms, septicemia, surgical site infections, acute kidney injury, respiratory failure and length of stay between POST and PRE time periods (Table 2). However, the POST cohort was associated with a significantly decreased rate of urinary tract infections (Table 2). Multivariate logistic regression analysis confirmed that the implementation of the restrictive transfusion policy (POST) was independently associated with a decreased risk of urinary tract infection after controlling for age, APACHE II score, and total amount of blood products transfused (Table 3).

## Discussion

When used properly, blood product transfusion can be lifesaving [12,13]. In addition, there is no doubt that anemia poses a risk to patient survival; however, in their current storage form, blood component transfusion does not alleviate this risk and carries its own significant inherent risks [12,13]. We have demonstrated that even in a SICU that does not operate under a fully ‘closed’ model in regards to patient orders, a restrictive protocol for PRBC and FFP transfusion can be implemented and successfully decrease overall blood product utilization. This occurred without an increase in morbidity or mortality, even in the setting of having a larger patient census and higher risk patients, as suggested by slightly increased ages and mean APACHE II scores. Both of the aforementioned co-factors are independently associated with worse outcomes in hospitalized populations [14,15]. Although a direct correlation cannot be made by our study, there was a significantly decreased rate of urinary tract infections associated with the implementation of this restrictive transfusion policy. In addition, at a minimal cost for implementing the protocol, we estimate we could have saved more than $130,000 per year.

It is clear that blood transfusion is associated with increased infections, morbidity and mortality in the surgical and critically ill populations [6]. A recent meta-analysis that reviewed the efficacy of human blood transfusion in the critically ill revealed that almost all studies (42 of 45) indicate that the risks of liberal PRBC transfusion outweigh the benefits; two of the remaining studies were neutral and the final remaining study demonstrated a benefit to transfusion in a subgroup of a single retrospective study (elderly patients with an acute myocardial infarction and a hematocrit <30%) [16]. The same report also determined that 17 of the 18 studies that appropriately reviewed mortality demonstrated that PRBC transfusion was an independent predictor of death. Finally, all of the 22 studies that analyzed PRBCs association with nosocomial infection concluded that blood transfusion was an independent risk factor for infection [9]. Recently, Juffermans *et al* determined that there was an association between the development secondary infections and PRBC transfusions [17].

Multiple reports have demonstrated that the utility of FFP administration is questionable for minor abnormalities in coagulation parameters [18,19]. In a prospective study, in all patients transfused with FFP, there was a 0.8% normalization of their INR [20]. There is a general lack of evidence of any known efficacy of FFP transfusion in clinical scenarios in which FFP are commonly prescribed [21]. FFP has been associated with a trend toward increasing mortality and an increased risk of acute lung injury [22].

Importantly for both PRBC and FFP, there are very few, if any, studies that have demonstrated a benefit from transfusion of these blood products [10,21]. In fact, FFP transfusion is notoriously unsuccessful in obtaining the goals of the transfusion, which are typically normalization of coagulation test and less bleeding [20,22,23]. However, protocolization can work to reduce inappropriate blood product transfusion, as has been demonstrated in trauma patients requiring neurosurgical interventions [24–26]. Previous studies have demonstrated a 75% reduction in use of FFP by protocolizing their plasma therapy [24]. Using stricter transfusion guidelines in septic shock patients, one study was able to show similar 90 day mortality and rates of ischemic events between higher and lower hemoglobin threshold [27].

The increased rates of UTI seen in this study may be due to multiple reasons. PRBC transfusions have been shown to have an association with the development of nosocomial infections in the initial phases of sepsis [17]. This may be due to various immunomodulations seen in transfusion patients including alloimmunization and tolerance induction [8]. These can then lead to decreased host response to antigens and subsequent infections. However, it is difficult to determine the relationship between decreased blood product usage and the decreased rate of UTIs in the POST group. There are multiple confounding variables including the definition used for UTI, which for our study was based on attending documentation and ICD-9 coding.

Even with all existing evidence about the general lack of benefit to PRBC and FFP transfusions, established physician practice habits can make enactment of best practice measures very difficult. One study demonstrated that even after all personnel agreed to only use FFP for agreed upon parameters, only 4% of the FFP ordered subsequent to the agreement appropriately met criteria [28]. Therefore, specific methodology in a ‘semi-open’ model unit was needed to reinforce education and maintain enforcement of the protocol. Our approach to this challenge was to have all attending surgical staff agree that (except in the case of the MTP) PRBC and FFP could only be ordered at the patient’s bedside after completing the form. This helped to further reinforce a change in clinical practice habits as well as often giving the SICU team a chance to communicate with the primary service to discuss the necessity of the transfusion.

To our knowledge, this is the first study to (1) report the impact of a restrictive protocol that is designed and implemented exclusively in the ICU setting, and (2) to include both PRBCs and FFP in the same change in practice. Although significant time and effort were required to create, educate staff about and apply this restrictive transfusion protocol, very little capital investment was required to successfully implement the restrictions. Blood products are a limited resource and transfusion of a single unit of PRBC is associated with a 10% increase in hospitalization costs [29].

At our institution, direct hospital costs for a single unit of leukoreduced PRBC and FFP at the time of the study were $183 and $51, respectively. During the POST time period, 323 PRBC and 131 FFP units were transfused, as opposed to 447 PRBC and 434 FFP units during the historical control period. Thus, 124 less PRBC units and 303 less FFP units were transfused to patients during the four month POST period. In addition, this was in the setting of a significantly greater average patient census than during the control time period. Extrapolated over one year, a conservative estimate of cost savings would be $130,000. One of the main weaknesses of this study is our inability to determine specific compliance data as well quality improvement (QI) data. The data collection sheets were used as part of patient improvement protocol and were required to be stored and subsequently disposed of in a HIPAA compliant manner (as they were not official parts of the patients chart) after weekly review by the ICU performance group. Feedback was given to those physicians who tried to order blood off protocol, as well as nursing staff who administered the blood ordered off protocol. Future studies should be prospective, rather than retrospective with historical controls, and collect and determine compliance and specific QI data.

## Conclusions

A protocol for restricting the use of multiple blood products can effectively and safely be instituted in a surgical intensive care unit, leading to a reduction in PRBC and FFP use in critically ill patients with no increase in morbidity or mortality. Additionally, restrictive transfusion policies may have a significant cost savings benefit when implemented in surgical intensive care unit settings.

## Supporting Information

S1 Dataset (XLSX)
